# Exploring the Efficacy of an Online Training Programme to Introduce Mental Health Recovery to Carers

**DOI:** 10.1007/s10597-023-01102-4

**Published:** 2023-02-24

**Authors:** Joanna Fox, Joannah Griffith, Anne Marie Smith

**Affiliations:** 1 Anglia Ruskin University, East Road, CB1 1PT Cambridge, UK; 2 University of Hertfordshire, Hatfield, UK

**Keywords:** Family carers, Care-givers, Mental health, Online training, Recovery approach

## Abstract

Family carers often support people with mental ill-health, however, there is a dearth of research on the importance of recovery to mental health carers. This article describes the delivery and qualitative evaluation of an online training programme on recovery to a group of eleven carers. The participants considered their understanding of the meaning of recovery, differentiating between its personal and clinical nature. They highlighted the importance of carer involvement in the service users’ professional support, alongside the need for carers to participate more widely in service development. Finally, the participants found the training useful in enabling them to recognise their own needs in a caring journey, particularly valuing its delivery by a service user and carer trainer. This study is limited by the small number of participants in this programme; however, this series of connected studies suggests its potential to be rolled out more widely, possibly embedded in Recovery Colleges.

## Introduction

Research connected to the care-giving experience in mental health has often focused on negative factors related to carer burden (Liu et al., 2020), or on expressed emotion in the relationship between the carers and the family member being supported (Amaresha & Venkatasubramanian, 2012). Moreover, studies often report the negative impact of caring on carers themselves (Poon et al., 2017; Carers UK, 2019). Despite this focus, there is now a growing evidence base which emphasises carers’ positive contribution to the wellbeing of their family member (Lauzier-Jobin & Houle, 2021) and reinforces the need to support them positively in their caring role (Fox et al., 2022; Waller et al., 2019). This article describes research which builds on a series of connected studies to evidence the usefulness of learning about recovery for carers (Fox et al., 2015, 2018, Fox et al., 2022) and aims to evaluate the effectiveness of participation in an online programme designed to introduce mental health recovery to carers to improve their sense of wellbeing and support them in their caring role.

## Literature Review

### Impact of Caring on Caregivers of People with Mental Ill-Health

Carers often experience a negative impact to their wellbeing from caring (Poon et al., 2017; Carers UK, 2019). In an Australian study, Poon et al. (2017) noted that a substantial percentage of carers experienced social isolation (28.6%), psychological distress (37.7%) and poorer quality of life than population norms; a situation also reflected in the UK (Carers UK, 2019). Furthermore, in a later study, Poon et al. (2018) suggested that carer’s perception of their loved one’s difficulties with socialization and independent living influenced the carers’ own social connectedness, grief and psychological health over time. Their findings suggested that carers’ perceptions of their family member’s ability to live well, or otherwise, with their condition, are a predictor of their own health and well-being. The persistently poor health and wellbeing of carers suggest a pressing need to enhance services that improve carers’ experiences, alongside that of the person they support (Poon et al., 2018). This background emphasises the need to support carers by reinforcing positive messages of hope in their role caring for a person with mental ill-health, such as those found in the recovery approach (Fox et al., 2022; Waller et al., 2019).

### The Importance of the Recovery Model to Carers

The recovery model is important for both service users and carers (Leamy et al., 2011; Jaiswal et al., 2020); it brings ideas of hope and optimism and reminds them of the importance of mental health recovery as a journey. The recovery concept was historically developed by service users (Coleman, 1999) to describe their self- management of their mental ill-health Personal recovery (Anthony, 1993) is distinct from clinical recovery in that it emphasises the non-linear trajectory of recovery. The elements of personal recovery are well-conceptualised in the model of CHIME, developed by Leamy et al. (2011), which represents an acronym to understand the service user’s recovery. CHIME incorporates five building blocks contributing to personal recovery: **c**onnectedness, **h**ope, **i**dentity, **m**eaning in life and **e**mpowerment. Despite its service user roots, some claim that the concept of recovery has become professionalised and lost its authenticity (De Ruysscher et al., 2019) becoming a service model that has been appropriated by professionals (Simpson et al., 2016; Slade, 2009). Additionally, it has become a service standard (DH, 2019) to guide professional practice and service outcomes; again, suggesting it has become detached from its roots. In this article, we understand this concept within the personal model of recovery that recognises that people with mental ill-health can lead a good life despite their symptoms (Anthony, 1993).

Although there is a growing evidence base of research undertaken with service users about recovery (Spaniol et al., 2002; Jaiswal et al., 2020), there is a dearth of research on the importance of recovery in the lives of carers. This research gap has persisted for over a decade as firstly defined in the literature (SRN, 2009, Marshall et al., 2013; Fox et al., 2015); although increasingly the importance of recovery is being understood in the context of mental health caring (Mundy, 2019; Marshall et al., 2013) concluded that further research was needed to better understand the nature of the relationship between carers’ recovery attitudes, wellness and other aspects of their caregiving experience, to facilitate the development of appropriate carer interventions and support in the future. Jacob et al. (2017) undertook a review which highlighted the gap in the current literature in relation to the views of mental health recovery among carers and service providers. To enable true partnership working to support the recovery process, and build a mental health system that upholds recovery, service users, carers and professionals need to understand recovery; hence research is required into the different perspectives held by these groups to support a shared understanding of the nature of recovery (Jacob et al., 2017).

Carers are important to the recovery of their family members and undertake many different roles in supporting them. Lauzier-Jobin and Houle (2021) highlighted five aspects to understanding the carers’ role in promoting the wellbeing of their family member. These included presence, companionship, emotional support, validation, and instrumental support. Moreover, the relationship between the carer and service user is often reciprocal (Allman et al., 2017) – with both the carer and the service user making positive contributions. As such, Allman et al. (2017) noted how the positive contributions young people experiencing first episode psychosis (FEP) offered to close relationships were often not recognised by their carers; the reasons for this were however not identified.

The recovery concept has become important to carers and has been used to denote their own journey of caring for someone with mental ill health. Mundy (2019), in a PhD study, used the CHIME components of personal recovery as a framework for understanding ‘family recovery’ narratives. **C**onnectedness centred on developing relationships within and outside of the family; **h**ope related to hopes that their family would come/stay together; **i**dentity and **m**eaning focused on the roles individual members played within the family; and **e**mpowerment related to how families facilitated the personal recovery of their loved one with psychosis and pursued their own recovery needs. This reflects a model of recovery for family members also identified in previous studies (Fox et al., 2015; Fox et al., 2022).

### Implementing Training on Recovery for Carers

In a series of connected studies, (Fox et al., 2015, 2018, Fox et al., 2022), the delivery of a training programme on recovery explored carers’ reactions to, and understanding of, the recovery journey. This programme was first delivered as a face-to-face course (Fox et al., 2015) then re-designed as a blended learning programme (Fox et al., 2018) and piloted in 2020 as a fully online programme (Fox et al., 2022). Originally, the 2020 course delivery was intended to be offered in a blended learning format, incorporating both face-to-face and online learning; however, delivery was forced to be fully online due to the emergence of the Covid-19 pandemic. This formed an unexpected innovation to the project.

The connected iterations of the programme led us to reflect on some learning points as we updated and re-designed the training for each delivery: travelling to face-to-face training could be very demanding for research participants and their geographical isolation could reduce their ability to engage fully with the course (Fox et al., 2015); peer support was a key part of this experience of face-to-face and online learning (Fox et al., 2015; Fox et al., 2022); participants appreciated the convenience of being able to attend training online (Fox et al., 2022); the ability to engage with online content and watch supplementary material was valuable if sessions were missed (Fox et al., 2022). Despite this, the content of the training, although updated and revised, reflected the successful overall themes developed in the initial training (Fox et al., 2015); although material was re-designed to fit an online model of delivery.

There are many advantages to online learning, although in academic situations many learners may lack the experience, knowledge, or confidence to access e-learning opportunities, or to access appropriate and effective technology (Young & Randall, 2014). Moreover, recent evaluations of different formats of online learning in Higher Education Institutions implemented during the pandemic (Bast, 2021; Sarkar et al., 2021) conclude that there are both benefits and barriers to online learning. For example, Almahasees et al. (2021) explored the implementation of online learning in Jordan during the period of the global pandemic; they reported that the challenges of adapting to online learning included the lack of interaction and motivation, technical and Internet issues, data privacy, and security, whilst the benefits included self-learning, low costs, convenience, and flexibility. Despite this mixed evidence, the pandemic situation in 2021/22, was constantly evolving, with the environment surrounding research governance changing continually in the UK; thus, this led us to focus on an online delivery of the programme for health and safety purposes, although the *intentional* utilisation of online packages of training for carers is a relatively recent development (Fox et al., 2018). The use of such formats needs to be carefully explored, as some participants may find the online delivery difficult, whilst for others it may be very convenient. Thus, in this current study, we built on the pilot (Fox et al., 2022) by recruiting a full sample of 11 participants, who expected the course to be delivered in a fully online format from the start and explored whether it was helpful for carers to learn about recovery through participation in an online programme.

## Methodology

We utilised a Participatory Action Research (PAR) model to support the development and design of the study. PAR emphasises a reflective cycle in which education transforms the participants through a process of learning and reflection (Freire, 1970). An advisory group, comprised of carers, service users, a peer trainer from the local Recovery College, and professionals, modified the study questionnaires and the online programme piloted in Fox et al. (2022). The advisory group met online on five occasions, utilising Microsoft Teams programme, a secure online communication platform. The study was underpinned by principles of coproduction as we sought to share power and decision-making across the members of the advisory group (Wallerstein et al., 2018).

Coproduction was a philosophy key to the development of this study. Coproduction (Rose, 2001; Bradley, 2015) is a practice that values and respects the perspectives of both service users and carers in the development of research, education, or training and thus underpinned the development and implementation of our study. Although service user involvement has a long history of implementation since the late 1990s (Rose, 2001), carer involvement is not so well-developed. Considering this, Bradley (2015) has underlined the importance of involving carers in coproducing services and in wider care planning and has noted that indirectly, coproduction can contribute to the recovery of persons being cared for. Coproduction values the involvement of people who use mental health services or non-working carers in the study equally, thus non-waged members of the advisory group were paid appropriately for their involvement in the research and participants in the training programme received a gift voucher to thank them for taking part in the study.

Participants completed online questionnaires before and after participating in the online training programme. Their socio-demographic data and their understanding of recovery were captured alongside their experiences of participation in the programme. These methods had been tested in the pilot completed in 2020, published in Fox et al. (2022). Additionally, participants were asked to respond to a scenario in a vignette about the fictious case study of ‘John’ and to consider what advice they would give to his parents, using the same scenario and questions both before and after the programme. John is portrayed as a young adult who was a trainee electrician who becomes mentally unwell and then enters a period of recovery. Hughes (1998) has highlighted the utility of vignettes as evaluation tools which can evidence changes in attitudes and behaviour as participants apply new learning to a scenario.

Three synchronous online sessions were delivered alongside two independent learning asynchronous online sessions. The asynchronous sessions were used to consolidate learning and moreover, if any of the participants missed the synchronous online teaching, they were able to view recorded audio and visual materials to reinforce learning. Online independent learning sessions were designed to be interactive and engaging, with material planned around the use of vignettes. The synchronous online sessions were co-delivered by the first author, an expert-by-experience with a diagnosis of schizophrenia and Associate Professor in social work, together with a carer-trainer. The content is shown in Table 1.

**Table 1:** Content of the training programme

Session	Content	Format
Pre-intervention contact	Information sheet/consent form Socio-demographic questionnaire: closed questions Questionnaire exploring response to case study: using open questions to clarify understanding of the meaning of recovery	Completed online
1	Introduction to recovery • Introduction to caring • Introduction to recovery • Recovery from JF’s (first author) personal viewpoint as an expert-by-experience	Synchronous Teams session
2	Carers’ assessment and recovery Using a case study which explores: • The stresses and joys of caring for a family member • Carers’ rights to a Carer’s Assessments by your local authority • Best practice in Carer’s Assessment	Completed online
3	A carer’s own journey of recovery • Working from a strengths model – the WRAP flower • What a recovery-oriented service might look like (CHIME model) • Introduction of a psychiatrist working in a recovery-oriented way: opportunities for Q&A	Synchronous Teams session
4	Mental health services and recovery Using a case study which introduces: • New professional roles and support processes which reflect a recovery approach to service provision. • A recovery-oriented Triangle of Care building on research completed by Fox (2018) • How services are provided through direct payments and individual budgets.	Completed online
5	The carer’s and service user’s journey • Exploring the differences between the service user’s expectations and the carer’s expectations of recovery • Considering carers’ own journey of living with and beyond the mental health issues of the service user • To answer any questions and explore any issues	Synchronous Teams session
Post-intervention	Questionnaire: Response to case study: using open questions to clarify changes in understanding of the meaning of recovery Open questions evaluating training program	Completed online

Participants were recruited to the course via convenience sampling. Information was shared on social media, via university newsletters, carers’ organisations and the local Recovery College networks. People could participate in the study if they self-identified as a carer of a person experiencing mental ill-health and the inclusion criteria did not require the person being cared for to be accessing services, or for the carers to be accessing support for themselves. We were approached by one carer supporting someone with an eating disorder; but experiences of eating disorders are very specific (Sepulveda et al., 2008) and the course was not directed to the needs of this caring population, so she did not join.

Data was analysed using thematic analysis (Braun & Clarke, 2022). The data was immediately pseudo-anonymised and analysed by the first author, JF. The themes were read multiple times, coded and recoded. A process of iterative movement between the literature and the data from the research allowed us to identify themes common to earlier research undertaken by the first author (Fox et al., 2015, Fox et al., 2022). The first author’s personal position in developing, delivering, and evaluating the program had the potential to lead to research bias. However, a process of reflexivity was utilized to counter any bias, and the themes were reviewed by other raters from the advisory group. JG checked for clarity in the presentation of the findings and the research themes.

### Ethical Approval

The study was given ethical approval by Anglia Ruskin University Faculty of Health, Education, Medicine and Social Care Ethics Committee (ESC-SREP-19-021). Prior to the study, the participants were asked to read an online information sheet and indicate their consent to take part in the research. The information sheet informed them of how their data would be used, that all information and quotations would be pseudo-anonymised on receipt, and that their quotations would be disseminated in study findings. Reassurances were given that all care would be taken to protect their identity, but that their quotations could lead to potential, if unlikely, identification. The participants were also emailed a copy of the information sheet.

We were aware that the participants might experience distress when discussing their caring situations; thus, we ensured that carer-trainer was available to support participants in an individual break-out room and that she participated in different small group discussions to provide ongoing assistance. From earlier iterations of the training (Fox et al., 2015; Fox et al., 2022) we were cognisant of the importance of peer support amongst the groups and thus ensured there was both space and time in the online programme to foster this sense of mutuality; although we acknowledge that this opportunity was limited through the online nature of the course.

## The Findings

Eleven participants attended the course. The sample was all female apart from one male. Ten participants were White British with one identifying as White Other. The participants cared for a variety of service users. The socio-demographics are identified in Table 2.

**Table 2:** Descriptive information for the carer and service user sample (n = 11)

	Carers		Service Users	
	Mean (*SD*)	Range	Mean (*SD*)	Range
**Age**	54 (*11.9*)	34–74	37 (*14.7*)	24–62
**Caring hours per week**	18 (9.8)	5–30		
	**Frequency**	**Percentage**	**Frequency**	**Percentage**
**Gender** Female/Male	10/1	91/9	10/1	91/9
**Marital status** Married Single Separated Divorced Widow/Widower	6 3 1 1	55 27 9 9	3 7 1	27 64 9
**Ethnicity** White British White Other	10 1	91 9	11	100
**Highest level of education** Secondary Tertiary/Further	4 7	36 64		
**Occupational status** Paid or self-employment Full-time Part-time Unemployed Student Retired due to age	6 1	55 9	7 1 1 2	64 9 9 18
**Living arrangements** Alone With husband/wife Together as a couple With parents	3 6 2	27 55 18	1 4 6	9 36 55
**Accommodation type** Owner Privately rented Rented from local authority/ housing association/ cooperative	6 3 2	55 27 18	5 2 4	46 18 36
**Caring for** Son Daughter Husband Father	6 1 3 1	55 9 27 9		
**Caring duties** Help in and around house Help in and around and outside house Help in and around and outside house plus childcare Help in and around and outside house plus other	2 6 1 1	18 55 9 9		
**Carer’s assessment** Yes No	3 8	27 73		
**Mental health issue onset** 1 year − 3 years More than 3 years ago			4 7	35 67

All participants who started the course attended most of the sessions and completed the pre- and post-course evaluation forms. Their attendance and levels of engagement with the asynchronous online material, ranging from 11 min to 19 h, are recorded in Table 3.

**Table 3:** Engagement with the course

Participant	Introductory session 25.04.22	Session 1 27.04.22	Session 2 11.05.22	Session 3 25.05.22	Engagement with online sessions
1	x	x	x	On holiday	2 h 06 min
2	x	x	x	On holiday	36 min
3	x	x	x	On holiday	1 h
4	x	x	x	x	11 min
5	x	x	x	x	25 min
6	x	Caring responsibility	x	x	50 min
7	x	x	x	Caring responsibility	3 h
8	x	x	x	x	18 min
9	x	x	x	On holiday	3 h 22 min
10	x	Caring responsibility	x	x	19 h 12 min
11	x	x	x	x	5 h 35 min

To capture the changes in the participants’ thoughts and reactions following participation in the training programme, data is presented in the findings section collected both before and after the training. Firstly, the findings revealed a definitive change in the participants’ understanding of the meaning of recovery before and after the course. They began to identify the potential of the recovery concept in generating hopefulness but recognised that mental ill-health might limit the recovery trajectory. The second theme consists of a consideration of the participants’ reflections on the factors which make up the recovery process. At the pre-course period, they believed that medication was essential for recovery; but following the course, they began to recognise the importance of other activities, such as reconnecting with friends and taking up volunteering opportunities, which may lead to a person’s social recovery. The third theme consists of the participants’ reflections on the centrality of the carer in the recovery process and the support they need to help them in this task.

### Understanding the Meaning of Recovery

This section presents the first of three themes which reveals the changes in the participants’ understanding of recovery both before and after the programme. Participants had limited understanding of the meaning of recovery before the course. Following the course, they acknowledged that their family member’s recovery may be limited due to their mental ill-health but realized the importance of focusing on hope and optimism.

In pre-course evaluations, participants exhibited a limited understanding of recovery including, “*how to help someone….obtain a better standard of living*” (Participant 2), “*learning, trying new approaches*” (Participant 5), “*a support package tailor made to the individual*” (Participant 6) “*to find a journey*” (Participant 10), “*to lead a fulfilling.life*” (Participant 11). However, evaluations tended to lack detail on recovery concepts such as hope, optimism, independence and self-management which participants address in the post-course questionnaires. Asked about the meaning of recovery, participant 5 reported:

*Learning to live with and manage your condition so that you can enjoy your life and be safe… Learning Exploring Trying new approaches Growing* (Participant 5 pre-course)

However, some expressed frustration at lack of improvement and grief for the hopes and dreams their family member may never achieve. Participant 8 seemed to be dispirited pre-course and expressed the feeling of having had “*very little support over the years” and “having to work out for ourselves how to manage*” (Participant 8). She continued:

*To be honest, I have struggled with the concept of recovery in relation to my husband’s mental health. He has experienced significant mental health issues since childhood and has to live with and manage this, rather than recovering from it.* (Participant 8 pre-course)

Participant 4 questioned the reality of recovery as she considered it might be unreachable; stating at the pre-course evaluation it might cause ‘*Too much pressure?’*

Following the training, participant 3 also acknowledged the sense of grief she experienced which contrasted with the positivity of the recovery concept. This made the recovery concept difficult to relate to, as captured here:

*Difficult to understand this concept when your loved one continues to be unwell and not able to fulfill their hopes and dreams. Difficult for a carer to fulfill the concept when your loved ones behaviour sometimes canot want support or help* (Participant 3 post-course)

Post-course, some carers understood recovery as a condition with limitations but one in which the service user could take control of their lives and live well; however, they would have to manage the illness and accept the limitations it brought. Participant 7 noted the importance of learning to self-manage the condition:

*The way I understand, is the process of coming to terms with the condition, understanding the triggers, the importance of medication and making small steps that will create a healthy routine which will allow the person to have a good quality of life* (Participant 7 post-course)

Participant 8 reflected on the importance of hope for her as a carer and that it was essential to reiterate this to their family member. When providing advice to the parents about John in the vignette, participant 8 considered at the post-course point, (in contrast to her quotation above):

*I think it is important that his parents don’t feel as though things will never change, and also important that they continue to support him by visiting if that is what he would like.* (Participant 8 post-course)

Others mention “*a better future*” and “*more of their [the service users’] own input*” (Participant 2), “*identity and meaning*” (Participant 3), “*meaningful life*” (Participant 5), “*positivity. purposeful life… improved relationships and connectedness*” (Participant 5), “*quality of life”, “opens up opportunities*” (Participant 7), “*learning to live with and beyond the illness*” (Participant 8). Thus, the participants recognized the positivity of recovery at the post-course point; this formed a significant shift in their thinking from the beginning of the study.

### The Factors Supporting the Recovery Process

#### Medication

The participants reflected on the importance of medication to their family member’s recovery at the start of the programme, but following the programme began to acknowledge the need for the service user to possess other strategies to achieve social recovery. This second theme highlights the factors that the participants identified as central to supporting the recovery process. When asked to consider the positive things about the recovery approach at this time, participant 2 stated:

*Continuation of medication, support of family and friends and being able to stay at home rather than going into hospital.* (Participant 2 pre-course)

Furthermore, in response to the vignette, positive outcomes were reported by participant 10 as relying on whether John would take medication or not.

*Relapse without medication an admission to hospital. Work temporary but can’t hold the job down, stress and anxiety the impact.* (Participant 10 pre-course)

It was interesting to note that following the intervention, two participants emphasised the importance of involving John in decision-making about his treatment choices and acknowledged the need to empower him in this, as exemplified by:

*Letting the service user make decisions about the important decisions in their life like their medication, employment etc....* (Participant 1 post-course)

Thus, medication was seen as central to the care of John, and a key component of any recovery plan. This treatment approach predominated at both the pre- and the post-course points.

#### Social Recovery

Although there was a focus on medication treatment to support both John’s recovery in the vignette, and the treatment of their own family member, many participants also acknowledged that other factors could contribute to recovery. The importance of alternative activities, which supported social recovery, were generated as another sub-theme in the data analysis. The findings underline the need for a service user to have a purposeful and meaningful life by taking part in activities which promote wellbeing.

Participant 8, after the intervention, stated his vision for John’s future presented in the vignette:

*I would like him to be able to maintain a job, socialise with his friends and feel a sense of purpose and enjoyment in his life.... This is what I would like to see for the person I care for.* (Participant 8 post-course)

Participant 11 believed that balancing medication needs and working towards accessing training with support from friends and carers *“will help John move along his recovery journey by being a support to him, providing positive experiences, giving him a routine and making his life happier and more meaningful”* (Participant 11 pre-course). For some participants, post-course, there was still a focus on the domestic context, sometimes more than wider sociability: “*home surroundings, calmer, with people who really care i.e. team and parents”* (Participant 2, post-course).

Post-course, one participant reflected on medication as a *tool* for supporting the process of social recovery.

*The medication could help to prevent the symptoms and paranoia so making it easier to make friends and improve relationships with family and friends. Support workers can help with getting into or back to work* (Participant 5 post-course)

Furthermore, reconnection with social networks was central to John recovering a life with purpose and meaning.

*it depends if John will carry on with healthy habits such as gym, seeing his friends and if he continues taking medication. If he takes his medication and continues the good habits he has strong chances to return to work and build his life.* (Participant 7 post-course)

A further shift in understanding post-course centres on the recovery concepts of interdependence and self-management. Post-course, carers say “*[recovery] can help to regain independence*” and “*take back control over [the service user’s] life*”, it can help with “*getting back to a purposeful and more content life*” (Participant 5), “*living as well as possible with a diagnosis*” (Participant 9), “*with more of their own input*” (Participant 2), and that it means service users “*being in control of [their] future, the future[they] want to have*” (Participant 11). The participants thus spoke more holistically about John’s recovery after the course, advocating the essence of social recovery in John’s life.

### Carer’s Roles and Needs

#### The act of Being a Carer

The role of carers was perceived as essential to the service user’s recovery. This is the third theme captured in the data at all points in the study. Some carers noted that they should put aside their discomfort and consider the needs of the service user. This was indicated when giving advice to John’s parents in the vignette

*Maybe also to try to put their feelings of discomfort to one side and try to see [the situation] more from how John is feeling.* (Participant 1 post-course).

Participants described their roles very much like a support to assist the service user to take part in the life they wanted. Moreover, they highlighted the importance of engaging in pleasant activities together.

*Meet on a regular basis to do pleasant activities together eg a walk, café, eat out, visit other relatives but small steps at a time, be supportive, be patient be brave* (Participant 5 pre-course)

At the pre-course point, participant 11 indicated that her role in recovery was being an *‘Encourager and enabler’*, however she emphasised that caring also had a monitoring feature on a day-to-day basis.

*I monitor his wellbeing on a day-to-day basis and remind him that the bad days will pass and better ones will come.* (Participant 11 pre-course)

A further aspect of the carer’s role was supporting their family member effectively as a ‘parent’, and not just as a ‘carer’. This manifested for participant 1 in not challenging her son’s behaviour in a negative way:

*I am just mum. I let my son guide me… I never challenge X in a negative way either.* (Participant 1 pre-course)

Despite this, there still seemed to be concerns after completion of the course over responsibility for medication and a nervousness about ‘not knowing’, perhaps depending on the stage of the illness and age of the service user, one participant saying, “*as without knowing it is impossible to look out for changes in the person’s behaviour*” (Participant 1), and another saying “*over the age of consent it is harder to gain information regarding…changes in medication, reliant on the individual passing information on, which may not happen*” (Participant 2). One suggests “*maybe more involvement for parents”* (Participant 1). Overall, the participants reported that carers played an important part in enabling recovery and consequently the participants stressed that carers should be involved in making treatment decisions alongside the service user. However, as participant 4 noted at the post-course evaluation point, mental health support ‘*sadly seems to be diminishing’*, which increases the carer’s tasks. They believed carer support and involvement was an essential aspect to any recovery plan.

#### Meeting the Needs of Carers

The participants in this study identified the importance of having their own needs met within the context of being a carer. This was a central theme found at both data collection periods. Participant 6 post-course highlighted the usefulness of a carers’ assessment,

*I would recommend the carers assessment … The carers assessment takes into consideration John, mum and dad feelings and includes them all in decision making which could bring them all closer together*

The participants recognised the importance of knowledge in their caring role. For example, when asked what advice she would give to John’s parents in the vignette, participant 11 stated:

*Knowledge is key to being able to understand your son’s needs and this can come from organisations such as Rethink, Mind etc etc. There is a lot of valuable information on the websites which can help you make sense of your son’s illness....* (Participant 11 post-course)

There is also the idea of on-going learning by the carers for the future, using practical elements of the course such as the *CHIME model* (Participant 3) “*tools we can draw on”* (Participant 10) “*a tool kit… and guidance”* (Participant 11), all of which seem to be valued as helpful and hopeful by carers. The participants felt that services played a part in supporting their needs as carers but acknowledged that they also had to take responsibility for managing their own learning about caring.

### Evaluation of Programme

Following the training there was an increased feeling of positivity about the care-giving role.

*Yes - … they helped me to recognise that actually the progress we had made was a lot more than I had realised, and gave me hope that even setbacks are just temporary, but we are on the right track.* (Participant 8 post course)

Moreover, despite the programme being delivered online, the participants appreciated the peer support generated from meeting with other carers in the programme. This was facilitated through online break out rooms in the Microsoft Teams application:

*All sessions helpful because it is all a learning experience and there is always something to learn. It is helpful to hear and learn about other people’s experiences* (Participant 5 post-course)

Two speakers were invited to the sessions: a psychiatrist who presented at the second session and a peer trainer from the recovery college who presented at the final session. The session with the psychiatrist was highly valued:

*The session with the psychiatrist for one hour It felt such a privilege to ask him whatever we wanted, He was so approachable and knowledgeable* (Participant 9 post-course)

The presentation from the recovery college was helpful “*for future reference”’* (participant 4). It was useful for the trainers to have multiple identities and able to share these with the participants. One trainer (the first author) was a service user, qualified and registered social worker, and an associate professor, and the second, a carer-trainer.

*It was so beneficial been taught by a service user and a carer trainer. Absolutely brilliant! I gained new perspective from them both about taken care of my dad but also taking care of myself thank you* (Participant 6 post-course)

One person valued the introduction of wellness tools such as Wellness Recovery Action Planning which was ‘*brilliant as it was such an easy way to demonstrate the process and explain it to others, including my son’* (*P*articipant11).

There was however some ambivalence about the use of the online supplementary asynchronous course. This is reflected in the diverse times that people engaged with it, as indicated in Table 3.

*I am still going through it and i find it very interesting* (Participant 7 post-course)

However, one found it hard to engage with the technology.

*Not very helpful. It was difficult to know where you were or what you had done. I once saved something to come back later but could not see it. Sometimes I think I might have done it twice. Confusing!* (Participant4 post-course)

Moreover, some appreciated that the course could be delivered online, whilst others would have valued more face-to-face content.

*I thought this enabled the sessions to be more inclusive and convenient. i wouldn’t have been able to commit to attending them in person.* (Participant 8 post-course)

Thus, the training programme was perceived as effective with some ambivalence at the fully online delivery of the programme.

## Discussion

### The Meaning of Recovery: Personal and Clinical Recovery

In this section, the significance of the study is highlighted. The first theme is the participants’ understanding of the meaning of recovery. It was difficult for participants to clearly relate to the meaning of personal recovery. For some it was unattainable and incomprehensible at the starting point of the course; but as the meaning became clearer during the programme, they expressed increased hope and optimism. The participants believed that it to be important to reinforce messages of hope to their family members when the service user was feeling low, distressed and dispirited. It was also important for them not to lose hope.

Throughout the course, the participants emphasised the perceived *necessity* for their family member to take medication to remain well. Despite this, some service users choose not to take, to cease or to reduce the medication they are prescribed or use it in a non-prescribed way to manage mental health symptoms (Watts et al., 2021; Keogh et al., 2022); and many service users do not tell their family members of this decision (Keogh et al., 2022) because of the potential negative reception. However, Keogh et al. (2022) reported that ceasing / reducing medication was rarely a reactive decision based on lack of insight but was often undertaken as a cost benefit analysis (Cappleman et al., 2015; Le Geyt et al., 2017), in which the choice to reduce / cease medication was considered in terms of its side effects and impact on their recovery journey. Moreover, the stance many carers hold, contrasts with the personal recovery model which advocates the importance of the service user self-managing their condition with or without the support of medication (Watts et al., 2021).

However, the views expressed by the participants tie in with research undertaken by Quaye and Rennoldson (2020), who investigated carers’ constructions of the meaning of recovery, and found that a medical interpretation of recovery predominated. Similarly, Gyamfi et al. (2020) noted that while some mental health professionals and students understood recovery to mean a personal process, others explained it as a clinical process. At the post-course period, the participants noted the importance of social recovery for their family member, which could be achieved through undertaking activities such as volunteering or re-connecting with friends. Despite referring to the concept of social recovery indirectly, the participants did not reference this concept directly in their evaluation.

### Recovery as a Journey for Carers

The participants emphasised that they had become more optimistic, seeing their caring as an active journey, noting how they had begun to recognise the journey they were traversing and to acknowledge the progress of the person they support. Some participants identified that it was important to realise that change was always possible (Participant 8) whilst another highlighted the sense of grief she was experiencing (Participant 3). However, despite this focus, none of the participants directly referred to the carer’s journey of recovery which has been emphasised in other studies (Fox et al., 2022; Mundy, 2019). By highlighting the carers’ own journey of recovery in the training, the participants could begin to recognise they progressed on their own recovery process. This had the effect of mediating the distress they experienced by reflecting on the progress they and their families were making. In the sessions, the groups were divided into smaller breakout rooms and shared their experiences with each other building on the power of peer support to build mutuality and trust.

The experience of grief in the carer’s journey ties in with research from Wyder et al. (2022) who explored the relevance of a model of family-based recovery with a group of family peer workers. The participants in Wyder et al’s (2022) study acknowledged that the family-based recovery model provided a useful framework for family engagement. However, it was noted in their study that a model of family-based recovery over-emphasised the positive dimensions of family recovery and did not reflect the ongoing distress and difficulties many family members continue to experience. Wyder et al. (2022) proposed the addition of a D dimension to the CHIME model to include the distress and difficulty evident for families. This recognises the sense of ongoing difficulty that many participants in our current study identified. However, as the participants identified, it is important for carers to support the recovery of their relative by emphasising messages of hope (SRN, 2009) and for service users to hear these reinforcements (Leamy et al., 2011).

### Carers Involvement: The Importance of Developing Effective Service Provision

Carers play an important role in the support of their family member (Lauzier-Jobin & Houle, 2021), often, as believed by the participants, in a more significant way than professionals. In the findings the participants noted very little about the influence of social workers or Community Psychiatric Nurses (CPNs), psychiatrists or psychologists on the care of their family member, as only support workers were mentioned. Indeed, it was noted that ‘*services are at crisis level’* (Participant 3 post-course). Professionals have a large part to play in facilitating the service user’s recovery. However, the participants feared that they would not be involved in their relative’s care, particularly when young adults became 18-years-old, and information was not automatically shared with the carer. Participant 11 highlighted the importance of all members of the care team to reinforce the same message of hope, including professionals, as other research has suggested (Salgado et al., 2010; Jackson-Blott et al., 2019); although she had received little professional input into the care of her family member. In a study undertaken before the pandemic, Simpson et al. (2016) found that care planning lacked a recovery focus and was often an administrative exercise, with a focus on risk avoidance rather than services based on therapeutic alliances between professional and service user and carer. Furthermore, Simpson et al. (2016) recognised an emergent cynicism amongst participants owing to recovery concepts and ideals being subverted by higher-order organisational needs, directives and ends. This suggests that even before the current crisis, the commitment to the delivery of recovery-oriented services may have been more rhetoric than reality.

Although the participants in the study had experienced very little support from services, they emphasised the importance of family-focussed care. Waller et al. (2019) explored the role of the family in the recovery journey of people experiencing mental ill-health from the perspectives of service users. However, despite acknowledging the importance of family members, the research participants in Waller et al’s (2019) study, noted that services did not engage with their family, despite them nominally receiving support from supposedly recovery-oriented services. Moreover, Ward et al. (2017) noted that mental health professionals found it difficult to articulate their understandings of Family Focused Practice (FFP) within a recovery framework. Nonetheless they were able to describe practices that embodied family-focused recovery. As demonstrated by Ward et al. (2017), there is a lack of research connecting FFP and recovery and how FFP is translated into practice, especially within a recovery framework. Furthermore, as the news media identifies, the experience of service provision has only worsened through the period of UK austerity and demand for services because of the COVID-19 pandemic from 2020 to 2022.

The participants in the study lacked some of the knowledge and language to express their desires for service provision. As far back as 2009, the importance of the Triangle of Care (Worthington & Rooney, 2009) was identified in modelling a partnership between the service user, professional and carer. Moreover, family-based practices such as Open Dialogue, originating in Finland (Seikkula, 2011) are advocated as important models to frame family involvement; as such, the effectiveness of Open Dialogue as a service model is now being evaluated in the UK. Other systemic practices, such as the Trialogue model (Mac Gabhann & Dunne, 2021; Amering et al., 2012), all support the increased involvement of family members in the recovery of people with mental ill health and provide an evidence-base for the inclusion of family members in the care of people with mental ill-health. Furthermore, the importance of developing family-focused practice in a *recovery context* is undeniable. Salgado et al. (2010) noted that attitudinal improvements for professionals following formal recovery training were not dependent on baseline levels of dispositional hope, therefore their study emphasised that institutions committed to recovery-oriented care should consider utilizing formal training. Jackson-Blott et al. (2019) undertook a quantitative literature review regarding recovery-oriented training programmes for mental health professionals. They concluded that whilst recovery training may have some utility in improving recovery-oriented staff outcomes, training needs to be provided as part of wider organisational change to ensure this translates into clinical practice. This underlines the importance of providing recovery-oriented and family-focused training to professionals in the care of the service user.

The training supported carers to understand the different roles professionals undertook and included information about carers’ rights to an assessment of their needs. Moreover, an online case study discussion highlighting reciprocal professional relationships enabled opportunities for the participants to explore these issues. Additionally, we invited a psychiatrist to join us as a guest speaker highlighting the use of recovery-oriented practice. The expertise was highly valued by the participants as he supported some to problem-solve issues about care and service provision for the person they cared for.

Thus, many participants felt a sense of increased positivity after the course and emphasised their role in the recovery of their family member. Involvement in their relative’s care, was key to any recovery plan, although there was a lack of professional input with many services neither being family-focused nor recovery-oriented. Innovative family practices such as Trialog (Mac Gabhann & Dunne, 2021; Amering et al., 2012) and Open Dialogue (Seikkula, 2011) need to be further developed with a focus on organisational recovery training for professionals (Salgado et al., 2010; Jackson-Blott et al., 2019).

### Evaluation of the Training

The participants emphasised the importance of peer support, learning from each other, and the role of the experts-by-experience in the training as essential to the success of the programme. The training was co-delivered by an Associate Professor, who is an expert-by-experience, and a carer-trainer. The Neon study (Rennick-Egglestone et al., 2020) highlights that it can be helpful for service users and carers to hear narratives of recovery told by other service users and carers. Moreover, a recent qualitative study (Ng et al., 2019) has identified three factors underpinning this sense of connection: comparison of self to narrator or narrative; feeling empathy for the narrator; and learning something from the narrative underlining the importance of the voice of the expert-by-experience in mental health interventions. This research reinforces this finding in our study.

However, it can be unusual for academics and professionals to share their experiences of mental ill-health. Lea et al. (2016) suggested that this reluctance to identify as a service user may be because this identity is perceived as problematic and disadvantageous and is highly stigmatised. Additionally, Schreur et al. (2015) highlighted the inclusion of service user involvement in clinical psychology training. They surmised that students learn from both the content and the process of service user involvement. The former incorporates the acquisition of knowledge on a conscious level, whilst the latter assumes that the process of learning together with service users implicitly influences the values and ethics of professionals. Thus, this evidence base reinforces the value we identified in our study for participants to hear the narratives of experts-by-experience.

Finally, the use of group-based training and experiential-led training is reported to be of great benefit in the design of training courses, found in our study. Jackson-Blott et al. (2019) noted that staff training interventions that provide group-based education on recovery principles and strategies appear to be successful. They noted that training programmes which included experiential learning had greater benefit than other formats of training, emphasising the importance of service-user involvement. Furthermore, the online learning format of this intervention supported previous findings that such an approach is effective because it can take place at times convenient to the participants (Johnson et al., 2010) and as reinforced by other studies (Mackay and Packenham, 2012) it focused on emphasising positive messages employing a mixture of online synchronous and asynchronous learning.

## Limitations

Firstly, this study was undertaken as a small-scale study with a small sample, therefore its applicability to wider contexts is limited. Moreover, all participants were from a White background, and there was only one male participant, with all others identifying as females. Participants supported a mixed group of family members, such as fathers (1), husbands (3) and grown-up children (7). Thus this study only included immediate family members. Despite this, some service users may not have immediate family and are supported by networks and extended contacts. Thus, future research should focus on supporting unpaid carers to participate who are not only immediate family members; although recruitment flyers in this current study invited all unpaid carers to participate, including people who identified as neighbours and friends.

Secondly, this study builds on the pilot programme delivered in 2020 (Fox, et al., 2022) and reflects earlier findings in a face-to-face delivery (Fox et al., 2015). This evidence continues to build the case for supporting carers to learn about recovery, and the utilisation of an online course, highlights its effectiveness in reaching people who are unable to travel. Participants in the study attended virtually from different areas of the UK, although the majority came from the Eastern region. Thus, geolocation is a limitation because carers in other places may have different experiences; however, the utilisation of online training can facilitate the inclusion of participants from a multitude of locations.

## Conclusion

This study sought to build on earlier work completed over the last 10 years (Fox et al., 2015, 2018; Fox et al., 2022) which explored whether it was helpful for carers to learn about recovery and to evaluate the effectiveness of an online programme in delivering this. An online training programme, utilising both synchronous and asynchronous learning, was delivered to a group of eleven carers.

The findings generated explored the learning of the carers about the meaning of recovery, the factors leading to recovery, the roles that carers occupy and the needs that they have. The online learning format of this intervention was found to be effective as it enabled training to be convenient at times chosen by the participant (Johnson et al., 2010); moreover, it focused on reinforcing positive messages (Mackay and Packenham, 2012) with a mixture of online synchronous and asynchronous learning.

The research highlights that further exploration is needed for carers to understand the use of medication in recovery, and to understand the importance of service user agency in such choices (Keogh et al., 2022). The carers in this study held strong convictions about the importance of service users taking medication as prescribed; thus, in future iterations of the course, it would be helpful to further explore with group participants the legitimacy of service users’ choices to cease / reduce medication and how for some, this is an active way in which they manage their recovery (Watts et al., 2021). Furthermore, this preceding series of connected studies (Fox et al., 2015, 2018; Fox et al., 2022) has suggested that it is important for carers to learn about recovery, and it can help them to care in a more hopeful and optimistic way. to develop this research, it will be important to roll out the course more widely, ideally embedded in a Recovery or Wellbeing College. This would ensure its greater accessibility to more participants and could promote the wellbeing and self-care of carers themselves.
